# Thrips Resistance Screening Is Coming of Age: Leaf Position and Ontogeny Are Important Determinants of Leaf-Based Resistance in Pepper

**DOI:** 10.3389/fpls.2019.00510

**Published:** 2019-04-24

**Authors:** Isabella G. S. Visschers, Janny L. Peters, Joep A. H. van de Vondervoort, Rick H. M. Hoogveld, Nicole M. van Dam

**Affiliations:** ^1^Department of Molecular Interaction Ecology, Institute for Water and Wetland Research, Radboud University, Nijmegen, Netherlands; ^2^Department of Molecular Plant Physiology, Institute for Water and Wetland Research, Radboud University, Nijmegen, Netherlands; ^3^German Centre for Integrative Biodiversity Research (iDiv) Halle-Jena-Leipzig, Leipzig, Germany; ^4^Institute of Biodiversity, Friedrich Schiller University Jena, Jena, Germany

**Keywords:** *Frankliniella occidentalis*, *Thrips tabaci*, *Capsicum*, pepper, insect resistance, crop breeding

## Abstract

*Capsicum* is a genus containing important crop species, many of which severely suffer from thrips infestation. Thrips feeding damages leaves and fruits, and often results in virus infections. Only a few insecticides are still effective against thrips, underlining the importance of finding natural resistance in crops. *Capsicum* is a perennial plant which is usually cultivated for several months, during which time the fruits are harvested. From the young vegetative stage to the mature fruit bearing stage, the plants are at risk to thrips infestation. Constitutive resistance to thrips over the entire ontogenetic development is therefore a key trait for a more sustainable and successful cultivation of the hot and sweet pepper. In addition to ontogeny, leaf position can affect the level of thrips resistance. Pest resistance levels are known to differ between young and old leaves. To our knowledge, no studies have explicitly considered ontogeny and leaf position when screening for constitutive resistance to thrips in *Capsicum*. In this study we analyze whether ontogeny and leaf position affect leaf-based resistance to *Frankliniella occidentalis* and *Thrips tabaci*, in 40 *Capsicum* accessions, comprising five different species. Our results show that resistance to both thrips species in *Capsicum* varies with ontogenetic stage. This variation in resistance among ontogenetic stages was not consistent among the accessions. However, accessions with constitutive resistance in both the flowering and fruit ripening stage could be identified. In addition, we found that thrips resistance is overall similar at different leaf positions within the ontogenetic stage. This implies that resistance mechanisms, such as defense compounds, are constitutively present at sufficient levels on all leaf positions. Finally, we found that resistance to *F. occidentalis* and resistance to *T. tabaci* were not correlated. This indicates that leaf-based resistance in *Capsicum* is thrips species-specific. Because of the variation in resistance over ontogeny, identifying *Capsicum* accessions with resistance over their entire lifespan is challenging. For resistance screening, accounting for leaf position may be less of a concern. To identify the defense mechanisms responsible for thrips resistance, it is important to further analyze and compare resistant and susceptible accessions.

## Introduction

Thrips are wide-spread piercing-sucking insects which are responsible for severe yield reduction in vegetable crops such as cucumber, strawberry, melon and pepper (Shipp et al., 2000; Park et al., 2007; Peng et al., 2011; Sampson and Kirk, 2013). Crops infected with thrips show stunted growth, leaf deformation and scarring of the fruits, leading to reduced yield and marketing quality (Welter et al., 1990; Tommasini and Maini, 1995; Shipp et al., 1998). In addition, they are an important vector of plant viruses, especially tospovirsuses (Whitfield et al., 2005; Riley et al., 2011; Rotenberg et al., 2015). Their positive thigmotactic nature, fast life cycle and parthenogenetic reproduction makes thrips difficult to combat. Thrips control is mostly achieved by integrated pest management (IPM) (Weintraub, 2007; Mouden et al., 2017), which combines chemical and biological strategies to grow healthy crops (Ehi-Eromosele et al., 2013). Pesticides have lost their effectiveness due to the emergence of resistant thrips populations (Bao et al., 2014; Li et al., 2016; Nazemi et al., 2016). Worldwide there are currently 175 documented cases of *Frankliniella occidentalis* populations and 112 cases of *Thrips tabaci* populations, which are resistant to insecticides (Arthropod Pesticide Resistance Database^1^, Michigan State University, East Lansing, MI, United States). In addition, the use of pesticides has been linked to bee colony disorder (Dively et al., 2015; Brandt et al., 2016), decline in insectivorous bird populations (Hallmann et al., 2014), and human health hazards (Kim et al., 2017). The successful application of IPM to minimize pesticide use is dependent on the presence of natural resistance in crop plants.

*Capsicum*, a genus in the nightshade family that includes hot and sweet pepper, can be severely damaged by thrips. Commonly grown species are *C. annuum* (chili and sweet pepper) and *C. chinense* (aromatic hot pepper). Nowadays, hot and sweet peppers are among the most produced crops, with an estimated production of 34.5 million tons worldwide (FAOSTAT; Data Productions Crops 2016^2^). Due to domestication, commercially grown hot and sweet pepper have lost their resistance to thrips and as a result *Capsicum* is infested by several thrips species (Talekar, 1991; Capinera, 2001; Ssemwogerere et al., 2013). Thrips species commonly found on *Capsicum* include *F. occidentalis* (western flower thrips), *Thrips palmi* (melon thrips), *Scritothrips dorsalis* (chilli thrips) and, to a lesser extent, *T. tabaci* (onion thrips) (Cannon et al., 2007; Weintraub, 2007; Walsh et al., 2012; Ssemwogerere et al., 2013). *F. occidentalis*, also known as Western Flower Thrips, is one of the most wide spread thrips species. It has successfully spread from America to Europe, Africa, Australia and Asia where it has established as a common pest (Tommasini and Maini, 1995; Kirk and Terry, 2003). It causes damage on leaves, flowers and developing fruits by feeding and egg deposition. Moreover, it can transmit at least five types of Tospoviruses (Riley et al., 2011). *T. palmi* and *S. dorsalis* are more problematic in tropical to subtropical regions (Cannon et al., 2007; Weintraub, 2007; Kumar et al., 2013). In Europe, they are observed only occasionally. Consequently, these thrips species are quarantine organisms in the EU (EPPO/CABI, 1998; Vierbergen and van der Gaag, 2009). *T. tabaci* was one of the first recognized vectors of the Tomato Spotted Wilt Virus (Pittman, 1927) and is the most serious pest in onion (Diaz-Montano et al., 2011). It is known to occur on a broad range of hosts, including *Capsicum*, and causes damage on the foliage (Ramakers, 1978; De Klerk and Ramakers, 1986; Walsh et al., 2012). Controlling thrips on *Capsicum* with pesticides is difficult and the identification of resistant accessions is necessary for successful and sustainable production of pepper in the future.

Various studies have explored sources of thrips resistance within the genus *Capsicum* (Fery and Schalk, 1991; Maris et al., 2003a; Maharijaya et al., 2011). Accessions resistant to thrips were characterized based on reduced preference, feeding or reproduction rates, or increased thrips mortality (Fery and Schalk, 1991; Maris et al., 2003b; Maris et al., 2004; Maharijaya et al., 2011). However, the various accessions tested do not always show consistent results throughout these studies. For example, the sweet pepper accession “Yolo Wonder” was reported to be resistant to *F. occidentalis* by Fery and Schalk (1991), but later identified as susceptible to the same thrips species by Maharijaya et al. (2011). These inconsistencies among studies might be explained by the fact that the accessions were screened at different ontogenetic stages. Because plant development is accompanied by physiological changes, resistance levels assessed in one ontogenetic stage cannot simply be extrapolated to other ontogenetic stages (Broekgaarden et al., 2012; Stout et al., 2013; Visschers et al., 2018a). *Capsicum* is a perennial plant, which bears fruits up to at least 160 days after the seedlings are planted (González-Dugo et al., 2007). An individual plant can suffer thrips damage from the early vegetative stage to the mature, fruit-bearing stage. Constitutive leaf-based resistance to thrips over the entire plant’s ontogenetic development is therefore a key trait for a more sustainable and successful cultivation of pepper. In addition to ontogeny, leaf position can affect the level of thrips resistance. Resistance to a given pest is known to differ between young and old leaves (van Dam et al., 1995; Visker et al., 2003; Alvarez et al., 2006; Gutbrodt et al., 2012). Moreover, whole plant assays have shown that within plant distribution of thrips is plant species- and cultivar- dependent (Reitz Stuart, 2002; Reay-Jones et al., 2017). Leaf position and ontogeny have, to our knowledge, not been included when screening for constitutive leaf-based resistance to thrips in *Capsicum*.

In this study we analyze whether ontogeny and leaf position affect leaf-based resistance to two different thrips species, *F. occidentalis* and *T. tabaci*, in *Capsicum*. Feeding damage was used as a parameter to determine leaf-based resistance levels, because this is a widely applied method for identifying resistant accessions (Koschier et al., 2002; Maris et al., 2003b; Leiss et al., 2009; Maharijaya et al., 2011). Moreover, feeding damage is positively correlated with thrips performance (Maharijaya et al., 2012), which makes it a good predictor for overall resistance. Our screening panel consisted of 40 accessions, including previously screened and novel *Capsicum annuum*, *Capsicum baccatum*, *Capsicum chinense*, *Capsicum pubescens* and *Capsicum frutescens* accessions. Using a high throughput leaf-disc based screening approach (Visschers et al., 2018a,b), the accessions were assessed for thrips resistance based on damage inflicted on leaf discs in three ontogenetic stages of the plant: the vegetative, flowering and fruiting stages. In the vegetative stage, only middle leaves were used for resistance screening, while in the reproductive stages apical and basal leaves were also included. Because the same accessions were screened for both *F. occidentalis* and *T. tabaci*, we could also directly correlate the resistance levels to these two species. A positive correlation might be indicative for a general resistance factor effective to multiple thrips species.

## Materials and Methods

### Plant Material

We used five *Capsicum* species, *C. annuum*, *C. chinense*, *C. baccatum*, *C. pubescens*, *C. frutescens* and a total of 40 accessions (Table 1). Several of these accessions have already been tested in Fery and Schalk (1991); Maris et al. (2003a), and Maharijaya et al. (2011) as being resistant or susceptible to thrips (Table 1). Seeds were obtained from the Centre for Genetic Resources (CGN), Wageningen University and Research Centre, Netherlands^3^. Accession “Hot fatalli” was obtained from East West Seed (Chiang Mai, Thailand).

**Table 1:** Overview of the forty *Capsicum* accessions used in this research.

Species	Accession code	Ru-code
*C. annuum*	CGN22151	1
*C. annuum*	CGN22830^I^	2
*C. annuum*	CGN22120^S^	3
*C. annuum*	CGN21543^R^	4
*C. annuum*	CGN21534	5
*C. annuum*	CGN23765^R^	6
*C. annuum*	CGN16913	7
*C. annuum*	CGN21550	8
*C. annuum*	CGN19226^I^	9
*C. annuum*	CGN19240	10
*C. annuum*	CGN20503^R^	11
*C. annuum*	CGN16922^I^	12
*C. annuum*	CGN19189^I^	13
*C. annuum*	CGN23222^RS^	14
*C. annuum*	CGN23098^IS^	15
*C. annuum*	CGN19239^R^	16
*C. annuum*	CGN23289^S^	17
*C. annuum*	CGN16835	18
*C. annuum*	CGN22151	19
*C. annuum*	CGN17202	20
*C. annuum*	CGN17227	21
*C. annuum*	CGN16975^R^	22
*C. chinense*	CGN17220	23
*C. chinense*	CGN22829^SR^	24
*C. chinense*	CGN22862^I^	25
*C. chinense*	Hot fatali	26
*C. chinense*	CGN16994^I^	27
*C. chinense*	CGN17004^S^	28
*C. chinense*	CGN21557^S^	29
*C. chinense*	CGN17222	30
*C. chinense*	CGN17219^S^	31
*C. chinense*	CGN16995^S^	32
*C. chinense*	CGN21469^R^	33
*C. frutescens*	CGN22839	34
*C. pubescens*	CGN22108	35
Unknown	VERGASA	36
Unknown	ROXY	37
Unknown	SNOOKER	38
*C. chinense*	CNG22798	39
*C. baccatum*	CGN21513	40

*Ru-codes are used for presentation in figures and tables. Letters following accession codes indicate previously identified resistance levels (^*R*^, resistant, ^*I*^, intermediate resistant and ^*S*^, susceptible) to thrips according to Fery and Schalk (1991); Maris et al. (2003a), and Maharijaya et al. (2011). Unknown in which ontogenetic stage thrips resistance was determined*.

Seeds were germinated in closed plastic cups (Ø 7 cm) on sterile glass beads (Ø 1 mm) in a climate cabinet (Snijders Labs, Tilburg, Netherlands) at L16:D8 light: dark regime and temperature set to 30°C/20°C (day/night). When the first two true leaves had developed, the seedlings were transplanted to pots (11 cm × 11 cm × 12 cm) containing potting soil (Potting soil 4, Horticoop, Bleiswijk, Netherlands). The pots were randomly placed on tables in a greenhouse, inside two insect-free net cages (Rovero 0.30 mm gauze, 7.50 m × 3 m × 2.75 m) at 16 h photoperiod and minimum temperatures set to 20°C/17°C (day/night). Natural light was supplemented with Greenpower lights (400 V/1000 W, Phillips, Amsterdam, Netherlands) when below 200 W m^-2^. Plants were inoculated with biological control agent, *Amblyseius swirskii* (Koppert Biological Systems, Berkel en Rodenrijs, Netherlands) every 4 to 6 weeks to minimize infection with thrips, whitefly and broad mite. Three to four individual plants per accession were maintained throughout the experiments. Sampling of leaves in the vegetative stage started 5 weeks after germination. After plants were sampled in the vegetative stage, they were transferred to larger containers (18 cm × Ø 13 cm) containing potting soil and 4 g L^-1^ Osmocote^^®^^ Exact Standard 3-4M (Everris, Waardenburg, Netherlands). Plants were watered with additional nutrient solution as necessary (1.8% Kristalon Label Blue, Yara, Grimsby, United Kingdom). For the flowering stage, leaves were collected after the first fully opened flowers had emerged on the plant. For the fruit ripening stage, leaves were collected when the first fruit started ripening and reached the breaker stage (fruit color changes from green to red). Experiments were conducted in two blocks, February 2015 through July 2015 for screening against *T. tabaci*, August 2015 through January 2016 for screening against *F. occidentalis*. Several accessions suffered from low levels of mite and aphid infestation in the reproductive stages (Supplementary Table S1). Plants were not sampled when yellow/white spots caused by spider mite feeding had occurred on leaves or when more than 10 aphids were observed on the plant.

### Insect Culture

To establish a colony, *F. occidentalis* and *T. tabaci* stock was obtained from Wageningen University, Netherlands. Cultures were kept in glass jars (11 cm × Ø 7.5 cm) with lids containing fine mesh gauze (45 μM polyester, Ø 6 cm) for aeration. *F. occidentalis* culture was kept on five fresh green beans (*Phaseolus vulgaris* L.) per jar and a 1.5 ml Eppendorf tube with a small amount of pollen grains (De Traay imkerij, Lelystad, Netherlands) to increase oviposition rates (Kirk, 1985; Anjum et al., 2012). *T. tabaci* was reared on two pieces of ±2 cm, Ø 3 cm *Allium ampeloprasum* var. porrum. Three layers of filtration paper were placed on the bottom to absorb excess moisture and to prevent the beans and leek from fouling. Thrips were transferred to clean jars weekly; beans and pieces of leek that were still looking fresh were transferred. The jars with thrips cultures were kept in separate climate cabinets (Economic Delux 432 L with TL lightning, Snijders Labs, Tilburg, Netherlands) at 25°C for *F. occidentalis* and 23°C for *T. tabaci* under a L16:D8 light regime. All experiments were performed using synchronized L1/L2 larvae that were starved for 24 h prior to experiments. Thrips colony rearing conditions and testing conditions were kept constant over time.

### No-Choice Leaf Disc Assay

**Figure 1:**
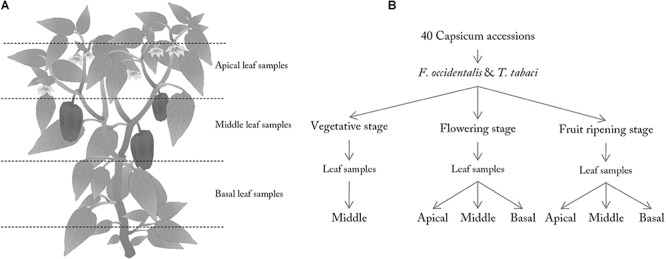
Schematic view of leaf sampling along the vertical axis of the plant **(A)** and overview of the experimental design **(B)**. Three plants per accession and life stage were sampled. Of each sampled plant two leaf discs per leaf position were tested for thrips damage (*n* = 6–12 leaf discs per accession and leaf position).

Leaf samples were collected in the greenhouse of the Radboud University as described by Visschers et al. (2018a). In the vegetative stage, leaf samples were selected in the middle of the plant’s vertical axis (Figure 1A). In the flowering and fruit ripening stage, apical (avoiding young, not fully developed leaves) and basal leaf samples (avoiding old discolored leaves) were taken in addition to middle leaf samples. For each plant, one leaf per position on the plant was collected for the no-choice assay (Figure 1B). Using a cork borer, four leaf discs (Ø 1.5 cm) were punched from the leaves, thereby avoiding the mid-vein for optimal image analysis (*n* = 4 leaf discs per leaf and leaf position on the plant). Each leaf disc was placed in a separate Petri dish on a drop of 1.5% slightly liquid agar (Sigma-Aldrich, United States) with the abaxial side up in the center of the Petri dish. For each sampled leaf, two of the four Petri dishes were inoculated with thrips. Thrips larvae were used for these experiments, since this stage is known to damage leaves the most. For inoculation, five synchronized L1/L2 thrips larvae were placed on the leaf disc using a small painting brush. The Petri dish was sealed with Parafilm and placed in the same climate cabinet as used for insect rearing. Petri dishes without thrips were directly sealed with Parafilm and used for correction during image analysis. After 48 h, the thrips were removed and digital images of treated and untreated leaf discs were acquired as describe by Visschers et al. (2018b). Leaf disc assays were conducted separately for each thrips species.

### Determination of Feeding Damage

**Figure 2:**
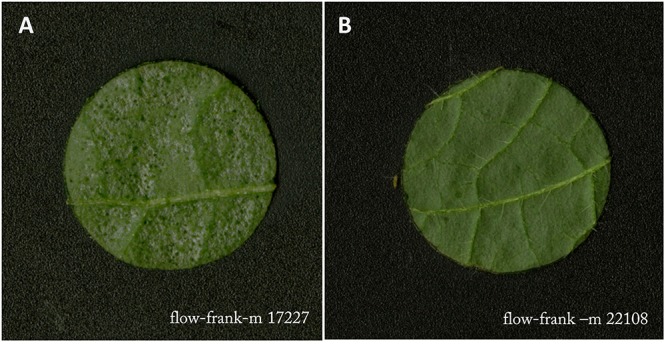
Example of severely damaged **(A)** and very little damaged **(B)** leaf disc by *Frankliniella occidentalis* in the flowering stage. Leaf samples were taken in the middle of the vertical axis of the plant.

Feeding damage included all discolorations of the leaf disc caused by thrips feeding, e.g., necrosis, dark green freshly damaged area and the typical silver leaf damage (Figure 2). Image processing and quantification of feeding damage in mm^2^ was performed using ImageJ Fiji (version 2.0.0 with Java 1.6.0_24) (Schindelin et al., 2012) and Ilastik (version 1.1.3) (Sommer et al., 2011) according to the protocol described by Visschers et al. (2018b). Ilastik was trained using four to six leaf discs per accession and life stage.

### Statistical Analysis

All statistical analyses were performed using R Version 1.0.153 (R Core Team, 2016). Statistical analyses were performed using non-parametric tests, since our data did not meet the requirements for parametric tests. Statistical analyses were preformed separately for each thrips species. Biological replicates were treated as independent replicates. Thrips damage (mm^2^) was transformed to ranks separately for each thrips species and ontogenetic stage in order to allow comparisons. This was necessary because the accessions differed in their ontogenetic development. Therefore, they were sampled at different time points, which caused random (temporal) variation in the amount of thrips damage. Accessions with equal scores were each assigned the same, average rank. For the *F. occidentalis* experiment, plants were ranked from 1 to 275 for both the vegetative and flowering stage; for the fruiting stage from 1 to 156, because some accessions became infested with greenhouse pests (see above) or did not set fruit. For the *T. tabaci* experiment, plants were ranked from 1 to 274 in the vegetative stage, in the flowering stage from 1 to 258 and in the fruit ripening stage from 1 to 209. Differences in resistance ranks among accessions within each ontogenetic stage were analyzed using the non-parametric Kruskal–Wallis H test. Differences among ontogenetic stage per accession were also analyzed using Kruskal–Wallis H test. Finally, effects of leaf position on thrips damage (in mm^2^) were assessed within each ontogenetic stage using Kruskal–Wallis H test. Significant effects were reported with alpha adjusted to 0.015 to correct for multiple comparisons.

Pairwise linear regression correlations of thrips damage between the ontogenetic stages and the leaf positions were performed using the “lm()” function from the Stats package in R. Correlation analyses among ontogenetic stages were based on the accession mean damage rank. Correlation analyses among leaf positions were based on accession-mean of mm^2^ damage per leaf position within ontogenetic stage. Correlation analyses among thrips species were based on accession mean of mm^2^ damage of middle leaves for each ontogenetic stage separately. Significant effects were reported with alpha set to 0.05.

## Results

In this study, 40 *Capsicum* accessions, comprising five species, were screened for resistance to two common thrips species. Using an objective leaf disc test, we analyzed whether ontogeny and leaf position affect resistance. Therefore, plants were screened over three ontogenetic stages and at three different leaf positions within the flowering and fruiting stages.

### Effects of Ontogeny on Thrips Damage

**Figure 3:**
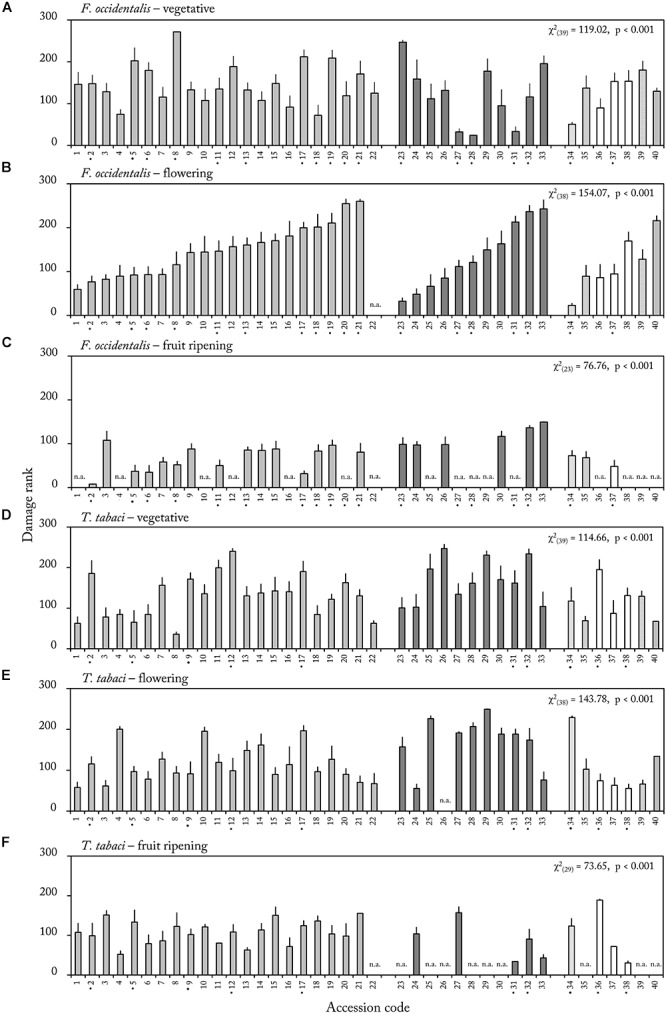
Mean (±SE) thrips damage rank of middle leave samples in the vegetative **(A,D)**, flowering **(B,E)** and fruit ripening stage **(C,F)** of 40 *Capsicum* accessions. Leaf discs were either exposed to *F. occidentalis* **(A–C)** or *T. tabaci* **(D–F)** (*n* = 4–12). Significant effect of accessions was observed for each life stage and thrips species (*p*-values of overall Kruskall–Wallis on effect of accession within ontogenetic stage). Accessions labeled with a • on the *X*-axis showed a significant effect of ontogeny on resistance rank (*p* < 0.015, see Supplementary Table S2 for details). Accession codes and corresponding CGN numbers can be found in Table 1. Different formatting of bars represents different *Capsicum* species. n.a. = not assessed due to mite and aphid infection.

Within each ontogenetic stage we analyzed whether thrips damage differed among the forty *Capsicum* accessions. In each ontogenetic stage, accessions significantly differed in thrips damage rank (*p* < 0.001 for each ontogenetic stage, Figure 3). This effect was observed for both thrips species (Figure 3). As a result, highly resistant and susceptible accessions could be identified in each ontogenetic stage. For example, in the flowering stage, accession CGN22839 (34) was only slightly damaged whereas accession CGN17227 (21) was severely damaged by *F. occidentalis* (mean thrips damage rank, 22.6 and 260, respectively, Figure 3B).

Next, we analyzed, within each accession, whether ontogeny significantly influenced thrips damage. For 55% of the accessions, based on the results of both thrips species, damage ranks were not consistent in the vegetative, flowering and fruit ripening stage (Figure 3 accessions marked by •, Supplementary Table S2). In 46% (*F. occidentalis*) and 25% (*T. tabaci*) of the accessions there was a significant effect of ontogenetic stage on thrips damage rank. For example, in accessions CGN17220 (23) and CGN17227 (21) we found a significant effect of ontogeny (χ^2^_(2)_ = 13.363, *p* = 0.001 and χ^2^_(2)_ = 12.877, *p* = 0.002, respectively) for resistance to *F. occidentalis*. Accession CGN17227 (21) was highly damaged in the flowering stage, but became less susceptible in the fruit ripening stage (mean thrips damage rank 260 and 80, respectively, Figure 3B,C). Accession CGN17220 (23) on the other hand, was one of the most resistant accession in the flowering stage, but among the most susceptible in the vegetative and fruit ripening stages (mean thrips damage rank 246.8, 32.2, and 98.4, respectively, in the vegetative, flowering and fruit ripening stage, Figure 3A–C).

To carefully explore this effect of ontogeny on thrips damage, we correlated mean damage ranks between ontogenetic stages. No significant correlation in damage rank was observed between most of the ontogenetic stages (Table 2). Only for *T. tabaci* we observed a positive correlation between damage ranks in the vegetative and flowering stage (*p* = 0.008, Table 2). Thrips damage, and thus resistance ranking, among the accessions is therefore dependent on ontogenetic stage.

**Table 2:** Pairwise linear regression correlation of thrips damage rank between the vegetative (veg), flowering (flow) and fruit ripening stage (fruit) for *F. occidentalis* and *T. tabaci*.

	*F. occidentalis*		*T. tabaci*	
	*R*^2^	*p*-value	*R*^2^	*p*-value
Veg – flow	<0.001	0.953	0.175	**0.008**
Veg – fruit	0.032	0.405	<0.001	0.992
Flow – fruit	0.132	0.087	0.005	0.719

*Coefficient of determination (*R*^*2*^) and the significance level (*p*) is given. Bold *p*-values (*p* < 0.05) indicate a significant positive correlation*.

### Effects of Leaf Position on Thrips Damage

**Figure 4:**
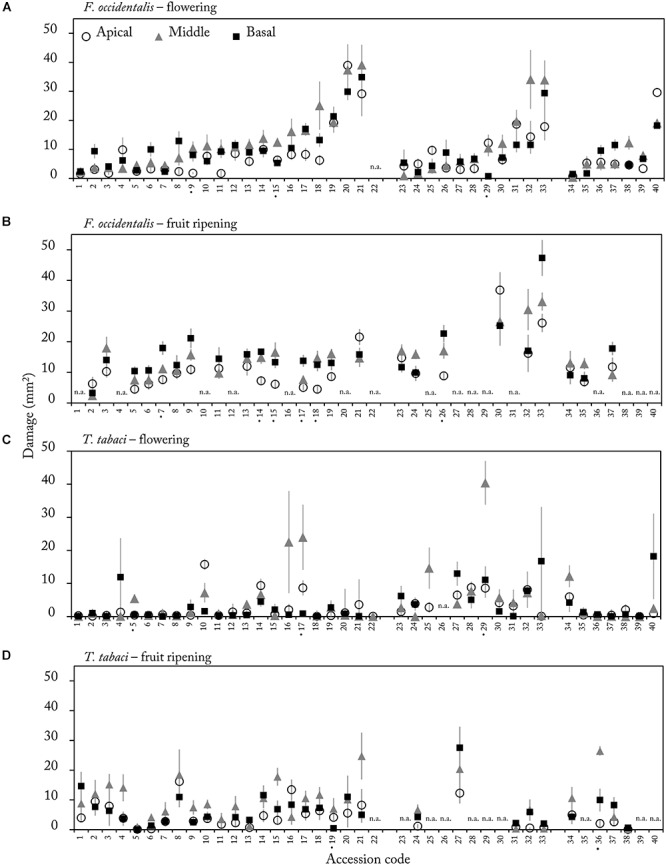
Mean (±SE) thrips damage (mm^2^) on apical (white), middle (gray) and basal (black) leaves in the flowering **(A,C)** and fruit ripening stage **(B,D)** on leaf discs (Ø 1.5 cm) of 40 *Capsicum* accession (*n* = 4–12). Leaf discs were either exposed to *F. occidentalis* **(A,B)** or *T. tabaci* **(C,D)**. Accession codes labeled with a • on the *X*-axis showed a significant effect of leaf position on damage (*p* < 0.015, Supplementary Table S3). Accession codes and corresponding CGN numbers can be found in Table 1. n.a. = not assessed due to mite and aphid infection.

We quantified thrips damage on leaf discs of apical, middle and basal leaves in the flowering and fruit ripening stage. Overall, middle leaves were damaged the most by both thrips species (mean thrips damage over all accessions for apical, middle and basal leaves were 8.5, 11.8, and 9.8 mm^2^, respectively). Significant effects of leaf position were only observed in a few accessions (Figure 4 accessions marked by •, Supplementary Table S3). Of these accessions only accession CGN23098 (15) demonstrated a significant effect of leaf position in both reproductive stages for *F. occidentalis* damage (flowering stage: χ^2^_(2)_ = 12.21, *p* = 0.002, and fruit ripening stage: χ^2^_(2)_ = 9.50, *p* = 0.009, Supplementary Table S3). Since the effect of leaf position was only observed in a few accessions, we tested whether feeding damage at a certain leaf position could be correlated to thrips damage at other leaf positions. Correlation analyses indeed confirmed that feeding damage was highly correlated between leaf positions. With *F. occidentalis*, these correlations were found in both the flowering and fruit ripening stages (Table 3). For *T. tabaci* a significant positive correlation was observed between basal and apical leaves and basal and middle leaves only in the fruit ripening stage. Thus, in most of the accessions thrips resistance scores are consistent at different leaf positions within an ontogenetic stage.

**Table 3:** Pairwise linear regression correlation of thrips damage between apical, middle and basal leaves in the flowering and fruit ripening stage for *F. occidentalis* and *T. tabaci*.

	Flowering stage				Fruit ripening stage			
	*F. occidentalis*		*T. tabaci*		*F. occidentalis*		*T. tabaci*	
	*R*^2^	*p*-value	*R*^2^	*p*-value	*R*^2^	*p*-value	*R*^2^	*p*-value
Apical - middle	0.628	**<0.001**	0.280	**<0.001**	0.487	**<0.001**	0.286	**0.002**
Apical - basal	0.608	**<0.001**	0.034	0.273	0.398	**<0.001**	0.349	**<0.001**
Middle - basal	0.695	**<0.001**	0.033	0.278	0.522	**<0.001**	0.285	**0.003**

*Coefficient of determination (*R*^*2*^) and the significance level (*p*) is given. Bold *p*-values (*p* < 0.05) indicate a significant positive correlation*.

### Correlation Between Resistance to *F. occidentalis* and *T. tabaci*

Finally, we tested whether thrips damage caused by *F. occidentalis* correlated to damage caused by *T. tabaci* in the vegetative, flowering and fruit ripening stage. We observed no significant correlation in mean damage ranks between these two thrips species. This effect was consistent for all three ontogenetic stages (Table 4).

**Table 4:** Pairwise linear regression correlation of damage rank between *F. occidentalis* and *T. tabaci* in the vegetative, flowering and fruit ripening stage.

	*R*^2^	*p*-value
Vegetative	0.025	0.331
Flowering	0.001	0.820
Fruit ripening	<0.001	0.904

*Coefficient of determination (*R*^2^) and the significance level (*p*) is given. No significant correlations were observed*.

## Discussion

In this study we provided evidence that leaf-based resistance to thrips changes over plant ontogeny. Although there was a strong effect of ontogeny, we identified several *C. annuum* and *C. chinense* accessions with consistent constitutive leaf-based resistance in the flowering and fruit ripening stage. We further demonstrate that when the plant is in a certain ontogenetic stage, resistance scores are mostly similar at different leaf positions. In addition, we found no correlation in damage rank for *F. occidentalis* and *T. tabaci*.

### Ontogenetic Stage Affects Leaf-Based Resistance to Thrips

Our results provide experimental evidence that leaf-based resistance to thrips in *Capsicum* varies with ontogenetic development. This means that it is important to control for ontogenetic stage when screening for resistance to thrips in *Capsicum*. Previous studies on other plant-insect systems have indicated the importance of ontogenetic stage when screening for resistance (Stout et al., 2013; Hoque and Avila-Sakar, 2015). Among crops, there is variation independent of ontogenetic stage. For example, field studies on cabbage revealed that *T. tabaci* preferred plants with developing cabbage heads over younger plants (Voorrips et al., 2008). Resistance to *F. occidentalis* in cucumber (*Cucumis sativus* L.), on the other hand, was not dependent on plant age (de Kogel et al., 1997a). However, in this study, it was not specified whether these differentially aged plants were also in different ontogenetic stages.

Our results show that the magnitude of the ontogenetic effect depends on the accession that is tested. In other words, we could not identify a general pattern for ontogenetic variation in leaf-based thrips resistance among the 40 *Capsicum* accessions. This is in line with a meta-analysis on the ontogeny of plant defense and herbivory, which also failed to find common patterns (Barton and Koricheva, 2010). This meta-analysis comprised 116 published studies reporting on ontogenetic patterns in defense traits and herbivory. It revealed that ontogenetic patterns were dependent on life form (woody, herbaceous, grass), type of herbivore (insect, mollusk, mammal) and defense trait (secondary chemistry, physical defense, tolerance) (Barton and Koricheva, 2010). Here we show that even within a group of congeneric species no general pattern in leaf-based resistance between ontogenetic stages to insects could be found. This means that identifying *Capsicum* accessions possessing constitutive leaf-based resistance over their entire lifespan is challenging. In *Capsicum* the reproductive stages are the most crucial for fruit set and development and last for several months. It can be argued that these stages are thus the most important for growers. On the other hand, natural enemies of thrips such as *Amblyseius swirskii* and *Orius laevigatus* establish when plants start to flower, due to availability of pollen (Goleva and Zebitz, 2013; Wong and Frank, 2013). Plants are therefore better protected by natural enemies in the reproductive stages, which makes leaf-based resistance in the vegetative stage more important. Natural leaf-based resistance in the vegetative stage combined with the application of natural enemies in the reproductive stage thus can contribute to successful implementation of IPM in *Capsicum*. As such, accessions such as CGN16994 (27) and CGN17004 (28), provide important leads for resistance breeding programs, because of their strong resistance in the vegetative stage. Our resistance measures are based on leaf disc-assays. Arguably, this may not reflect resistance on the whole plant level, where flowers are available. However, previous studies have shown that thrips damage observed in leaf disc assays and whole plant assays yield comparable results (Maharijaya et al., 2011). Leaf disc assays, especially when combined with computerized data acquisition, thus may be a cost-effective method to quickly identify suitable accessions for breeding thrips resistant crops. Nevertheless, whole plant screening assays including the monitoring of population development can provide additional information on the level of antibiosis in these resistant accessions, especially when plants have entered the generative stage.

Additionally, we have shown that both *C. annuum* and *C. chinense* include accessions with constitutive leaf-based resistance in the flowering and fruit ripening stages such as accession CGN22151 (1), CGN22830 (2) and CGN23765 (6). These accessions provide interesting targets for identifying resistance mechanisms to thrips. Each *Capsicum* species might possess its own unique set of metabolites that confers resistance to thrips. In the two closely related *Solanum* species, *S. galapagense* and *S. cheesmaniaeare*, metabolomics profiles were distinctively different (Vosman et al., 2018). Based on these studies, we expect metabolomics profiles and consequentially metabolites conferring resistance to differ between *C. annuum* and *C. chinense* as well. As both species can be hybridized, heritable (chemical) resistance may be combined to create a multilayered thrips defense which lasts over the plant’s reproductive lifetime (Zewdie and Bosland, 2000).

### Leaf Position Does Not Affect Thrips Resistance

Our study also shows that thrips resistance within ontogenetic stage is mostly constant at different leaf positions. In previous studies on within-plant distribution of insect feeding, leaf position was found to affect resistance to thrips. In cucumber (*C. sativus*), leaf position had a significant effect on reproduction of *F. occidentalis*, and this effect was different among accessions (de Kogel et al., 1997a,b). Similarly, in cotton, thrips abundance within the plant was affected by leaf position and this effect was dependent on the tested cultivar (Reay-Jones et al., 2017). We show that in *Capsicum* leaf position does not affect resistance to *F. occidentalis* or *T. tabaci*. This suggests that resistance mechanisms, such as defense compounds, are constitutively produced in leaves over the whole plant, independent of leaf position. In other studies, presence of defense compounds in apical and basal leaves have shown contrasting patterns. In maize (*Zea mays* L.), the defense compound 1,4-benzoxazin-3-one, an antifeedant and toxic compound to a wide range of herbivores (Niemeyer, 2009), was found to be the lower in young leaves than in old leaves (Kohler et al., 2015). However, in *Cynoglossum officinale* (Houndstongue) opposite patterns were observed. Young leaves contained up to 190 times higher levels of pyrrolizidine alkaloids, which are deterrent to generalists’ herbivores (van Dam et al., 1994). In *Capsicum*, capsianoside III-2, a linear diterpene glycoside with a hydroxygeranyllinalool skeleton, has been linked to resistance to thrips in a cross between a resistant *C. annuum* and susceptible *C. chinense* (Maharijaya et al., 2018). The distribution of this compound over different leaf positions is not known, though it has been found in the fruits (Lee et al., 2006). In *Nicotiana attenuata* (coyote tabacco), capsianosides provided an effective direct defense against *Manduca sexta* (tobacco hornworm) (Heiling et al., 2010). Interestingly, the allocation of this defense compound in *N. attenuata* changes with leaf position, with the highest concentrations found in young leaves and reproductive tissues (Heiling et al., 2010). Our results show that resistance to thrips is not affected by leaf position. Therefore, it is likely that thrips-related defense compounds, such as capsianosides, are either homogeneously allocated or, in resistant accessions, present at sufficient levels at each position to reduce thrips feeding.

### Resistance in *Capsicum* Is Thrips Species-Specific

We observed no correlation between damage caused by *F. occidentalis* and *T. tabaci*. This is in contrast with the study of Maharijaya et al. (2011). They determined resistance to *Thrips parvispinu*s and *F. occidentalis* in 32 *Capsicum* accessions and found that resistance to these two thrips species was positively correlated. They used this as evidence to argue that there are global resistance mechanisms to thrips (Maharijaya et al., 2011). Our results, which were obtained over a broader panel of accessions and at multiple ontogenetic time points, rather suggest that resistance is thrips species-specific. This makes it less likely that there is a general resistance factor to all thrips species in *Capsicum*. However, this does not preclude that a single accession cannot be resistant to multiple thrips species. A study on resistance to several aphid species in *Medicago truncatula* (barrel clover) showed that one of the tested accessions provided resistance to three aphid species, while failing to provide resistance to two other aphid species (Gao et al., 2007). Their findings indicate some level of aphid species-specificity in the plant’s resistance mechanism and a strong influence of the plants genetic background on this specificity. Similar aspects might play a role in resistance mechanisms to thrips in *Capsicum*. Resistance to *T. tabaci* might be driven by a different genetic mechanism than resistance to *F. occidentalis* and *T. parvispinus*. Such aspects might partially explain the dissimilarities between our findings and those of Maharijaya et al. (2011).

In order to identify chemical traits conferring resistance to thrips, accessions with contrasting effects on thrips damage, such as CGN21469 (33), which is susceptible to *F. occidentalis* and moderately resistant to *T. tabaci*, might be interesting models for identifying defense compounds against different thrips species. This can be achieved, for example, by fractionation-driven bioassays (Rimando et al., 2001; Tsao et al., 2005; Wahyuni et al., 2013). This method integrates the process of separation of bio-active compounds by fractionation methods (e.g., liquid or gas chromatography) with results obtained from biological testing, e.g., thrips performance. In *Eurythrina crista-galli* this method was successfully applied to identify botanical insecticides for cotton aphids (*Aphis gossypii*) (Wang et al., 2018). In addition, this method allowed the identification of a novel unsaturated isobutylamide, providing resistance to *F. occidentalis* in *Chrysanthemum* (Tsao et al., 2005).

Altogether, our findings show that identifying accessions with broad scale leaf-based resistance to thrips is a challenging endeavor, even with plants growing under controlled greenhouse conditions. More knowledge on thrips species-specific defense mechanisms might provide important clues for more targeted identification of broad scale thrips resistance.

## Author Contributions

IV, JP, and NvD contributed conception and design of the study. JP and NvD revised it critically for important intellectual content and provided approval for publication of the content. IV provided substantial contributions to the acquisition, analysis and interpretation of the data. JvdV and RH provided substanial contributions to acquisition and analysis of the data.

## Conflict of Interest Statement

The authors declare that the research was conducted in the absence of any commercial or financial relationships that could be construed as a potential conflict of interest.

## Footnotes

1 https://www.pesticideresistance.org/

2 http://www.fao.org/faostat/en/\#data/QC

3 http://cgngenis.wur.nl/
