# Supplementary material for: Thrips Resistance Screening Is Coming of Age: Leaf Position and Ontogeny Are Important Determinants of Leaf-Based Resistance in Pepper
Source: Front Plant Sci. 2019 Apr 24;10:510. doi: 10.3389/fpls.2019.00510 (PMC6491929; doi:10.3389/fpls.2019.00510)
Supplement: Supplementary file 1 [file Table_1.docx]

Table S1. Overview of *Capsicum* accessions that were screened in several blocks from February 2015 through July 2015 for screening against *Thrips tabaci*, August 2015 through January 2016 for screening against *Frankliniella occidentalis* in no-choice bio-assay in the vegetative, flowering and fruit ripening stage.

| **Ru-code** | **Sample block *F. occidentalis*** | | | **Sample block *T. tabaci*** | | | |
| --- | --- | --- | --- | --- | --- | --- | --- |
|  | **Vegetative** | **Flowering** | **Fruit ripening** |  | **Vegetative** | **Flowering** | **Fruit ripening** |
| 34 | 2 | 8 | 13 |  | 2 | 7 | 12 |
| 23 | 1 | 7 | 13 |  | 1 | 5 | n.a. |
| 24 | 1 | 8 | 13 |  | 1 | 6 | 9 |
| 1 | 4 | 7 | n.a. |  | 1 | 4 | 9 |
| 25 | 4 | 7 | n.a. |  | 2 | 8 | n.a. |
| 2 | 1 | 8 | 12 |  | 1 | 5 | 12 |
| 3 | 2 | 7 | 12 |  | 2 | 5 | 12 |
| 26 | 4 | 8 | 13 |  | 3 | n.a. | n.a. |
| 36 | 1 | 6 | n.a. |  | 1 | 5 | 9 |
| 35 | 3 | 8 | 13 |  | 3 | 5 | n.a. |
| 4 | 1 | 8 | n.a. |  | 1 | 4 | 9 |
| 5 | 2 | 8 | 13 |  | 2 | 6 | n.a. |
| 6 | 1 | 7 | 13 |  | 2 | 4 | 10 |
| 7 | 2 | 6 | 10 |  | 2 | 4 | 9 |
| 37 | 1 | 6 | 10 |  | 1 | 4 | 9 |
| 27 | 2 | 8 | n.a. |  | 2 | 6 | 11 |
| 8 | 1 | 6 | 10 |  | 1 | 5 | 9 |
| 28 | 1 | 8 | n.a. |  | 1 | 6 | n.a. |
| 39 | 3 | 8 | n.a. |  | 3 | 5 | n.a. |
| 9 | 2 | 6 | 12 |  | 2 | 4 | 10 |
| 10 | 1 | 6 | n.a. |  | 1 | 7 | 11 |
| 11 | 1 | 8 | 13 |  | 1 | 8 | 12 |
| 29 | 1 | 8 | n.a. |  | 1 | 8 | n.a. |
| 12 | 2 | 6 | n.a. |  | 2 | 4 | 10 |
| 13 | 2 | 7 | 13 |  | 2 | 6 | 12 |
| 30 | 2 | 7 | 13 |  | 2 | 8 | n.a. |
| 14 | 1 | 7 | 12 |  | 1 | 8 | 12 |
| 38 | 1 | 7 | n.a. |  | 1 | 5 | 9 |
| 15 | 1 | 6 | 12 |  | 1 | 4 | 10 |
| 16 | 1 | 6 | n.a. |  | 1 | 4 | 9 |
| 17 | 2 | 6 | 10 |  | 2 | 4 | 9 |
| 18 | 3 | 6 | 11 |  | 3 | 4 | 10 |
| 19 | 4 | 7 | 10 |  | 1 | 4 | n.a. |
| 31 | 1 | 8 | n.a. |  | 1 | 6 | 12 |
| 40 | 3 | 6 | n.a. |  | 3 | 7 | n.a. |
| 32 | 1 | 8 | 13 |  | 1 | 6 | 12 |
| 33 | 2 | 6 | 13 |  | 2 | 4 | 10 |
| 20 | 2 | 8 | n.a. |  | 1 | 4 | 9 |
| 21 | 1 | 6 | 11 |  | 1 | 5 | 9 |
| 22 | 3 | n.a. | n.a. |  | 3 | 8 | n.a. |
| n.a. = not accessed due to *Aphidoidea* sp. or *Tetranychus urticae* (Two spotted spider mite) infection | | | | | | | |

Table S2. Kruskall-Wallis H test on the effect of ontogeny on thrips damage in 40 *Capsicum* accessions for *F. occidentalis* and *T. tabaci*. Bold P-values (< 0.015) indicate a significant effect of ontogenetic stage within an accession.

|  | ***F. occidentalis*** | | |  | ***T. tabaci*** | | |  |
| --- | --- | --- | --- | --- | --- | --- | --- | --- |
| **Ru-code** | **χ^2^** | **df** | **P-value** |  | **χ^2^** | **df** | **P-value** |  |
| 34 | 10.331 | 2 | **0.006** |  | 11.031 | 2 | **0.004** |  |
| 23 | 13.363 | 2 | **0.001** |  | 3.415 | 1 | 0.065 |  |
| 24 | 3.618 | 2 | 0.164 |  | 2.690 | 2 | 0.261 |  |
| 1 | 5.026 | 1 | 0.025 |  | 2.674 | 2 | 0.263 |  |
| 25 | 0.650 | 1 | 0.420 |  | 0.006 | 1 | 0.936 |  |
| 2 | 13.168 | 2 | **0.001** |  | 8.611 | 2 | **0.013** |  |
| 3 | 4.294 | 2 | 0.117 |  | 4.972 | 2 | 0.083 |  |
| 26 | 1.761 | 2 | 0.415 |  |  | n.a. |  |  |
| 36 | 0.103 | 1 | 0.748 |  | 9.840 | 2 | **0.007** |  |
| 35 | 3.199 | 2 | 0.202 |  | 0.235 | 1 | 0.628 |  |
| 4 | 0.184 | 1 | 0.668 |  | 3.458 | 2 | 0.177 |  |
| 5 | 10.187 | 2 | **0.006** |  | 11.799 | 2 | **0.003** |  |
| 6 | 14.429 | 2 | **0.001** |  | 0.056 | 2 | 0.972 |  |
| 7 | 4.979 | 2 | 0.083 |  | 3.694 | 2 | 0.158 |  |
| 37 | 8.854 | 2 | **0.011** |  | 0.977 | 2 | 0.614 |  |
| 27 | 8.308 | 1 | **0.004** |  | 2.435 | 2 | 0.300 |  |
| 8 | 12.756 | 2 | **0.002** |  | 4.452 | 2 | 0.108 |  |
| 28 | 8.307 | 1 | **0.004** |  | 2.084 | 1 | 0.149 |  |
| 39 | 2.084 | 1 | 0.149 |  | 5.398 | 1 | 0.020 |  |
| 9 | 3.557 | 2 | 0.169 |  | 9.064 | 2 | **0.011** |  |
| 10 | 0.410 | 1 | 0.522 |  | 6.038 | 2 | 0.049 |  |
| 11 | 8.503 | 2 | **0.014** |  | 7.695 | 2 | 0.021 |  |
| 29 | 0.410 | 1 | 0.522 |  | 0.521 | 1 | 0.470 |  |
| 12 | 1.2564 | 1 | 0.262 |  | 9.638 | 2 | **0.008** |  |
| 13 | 10.842 | 2 | **0.004** |  | 3.356 | 2 | 0.187 |  |
| 30 | 3.173 | 2 | 0.205 |  | 0 | 1 | 1 |  |
| 14 | 5.725 | 2 | 0.057 |  | 2.677 | 2 | 0.262 |  |
| 38 | 0.231 | 1 | 0.631 |  | 12.652 | 2 | **0.002** |  |
| 15 | 8.011 | 2 | 0.0182 |  | 5.338 | 2 | 0.069 |  |
| 16 | 3.403 | 1 | 0.065 |  | 3.572 | 2 | 0.168 |  |
| 17 | 21.180 | 2 | **0.000** |  | 8.841 | 2 | **0.012** |  |
| 18 | 8.887 | 2 | **0.011** |  | 3.970 | 2 | 0.137 |  |
| 19 | 9.871 | 2 | **0.007** |  | 0.825 | 2 | 0.662 |  |
| 31 | 8.337 | 1 | **0.004** |  | 11.047 | 2 | **0.004** |  |
| 40 | 4.000 | 1 | 0.046 |  | 3.158 | 1 | 0.076 |  |
| 32 | 9.169 | 2 | **0.010** |  | 11.626 | 2 | **0.003** |  |
| 33 | 7.036 | 2 | 0.030 |  | 2.8155 | 2 | 0.245 |  |
| 20 | 7.410 | 1 | **0.007** |  | 3.452 | 2 | 0.178 |  |
| 21 | 12.877 | 2 | **0.002** |  | 7.536 | 2 | 0.023 |  |
| 22 |  | n.a. |  |  | 0.107 | 1 | 0.743 |  |

Table S3. Kruskall-Wallis H test on the effect of leaf position on thrips damage in the flowering and fruit ripening stage for *F. occidentalis* and *T. tabaci*. Bold P-values (< 0.015) indicate a significant effect of leaf position within an accession.

|  | ***F. occidentalis*** | | | | | | | | |  | ***T. tabaci*** | | | | | | |
| --- | --- | --- | --- | --- | --- | --- | --- | --- | --- | --- | --- | --- | --- | --- | --- | --- | --- |
|  | **Flowering stage** | | |  | **Fruit ripening stage** | | | | |  | **Flowering stage** | | |  | **Fruit ripening stage** | | |
| **Ru-code** | **χ^2^** | **df** | **P-value** |  | **χ^2^** | | **df** | | **P-value** |  | **χ^2^** | **df** | **P-value** |  | **χ^2^** | **df** | **P-value** |
| 34 | 0.56 | 2 | 0.756 |  | 2.35 | | 2 | | 0.309 |  | 6.49 | 2 | 0.039 |  | 2.33 | 2 | 0.312 |
| 23 | 1.09 | 2 | 0.578 |  | 3.95 | | 2 | | 0.138 |  | 4.59 | 2 | 0.101 |  | n.a. |  |  |
| 24 | 2.06 | 2 | 0.358 |  | 4.67 | | 2 | | 0.097 |  | 8.84 | 2 | 0.012 |  | 5.78 | 2 | 0.055 |
| 1 | 0.72 | 2 | 0.697 |  | n.a. | |  | |  |  | 0.93 | 2 | 0.627 |  | 3.69 | 2 | 0.158 |
| 25 | 4.44 | 2 | 0.109 |  | n.a. | |  | |  |  | 5.04 | 1 | 0.025 |  | n.a. |  |  |
| 2 | 4.89 | 2 | 0.087 |  | 1.85 | | 2 | | 0.397 |  | 4.76 | 2 | 0.092 |  | 0.95 | 2 | 0.621 |
| 3 | 5.51 | 2 | 0.063 |  | 4.46 | | 2 | | 0.107 |  | 1.04 | 2 | 0.595 |  | 2.98 | 2 | 0.225 |
| 26 | 1.00 | 2 | 0.605 |  | 10.40 | | 2 | | **0.005** |  | n.a. |  |  |  | n.a. |  |  |
| 36 | 3.56 | 2 | 0.169 |  | n.a. | |  | |  |  | 1.79 | 2 | 0.409 |  | 11.39 | 2 | **0.003** |
| 35 | 1.98 | 2 | 0.372 |  | 4.92 | | 2 | | 0.085 |  | 0.14 | 2 | 0.930 |  | n.a. |  |  |
| 4 | 4.09 | 3 | 0.252 |  | n.a. | |  | |  |  | 0.47 | 2 | 0.791 |  | 4.11 | 2 | 0.128 |
| 5 | 2.38 | 2 | 0.304 |  | 5.29 | | 2 | | 0.071 |  | 11.43 | 2 | **0.003** |  | 1.92 | 2 | 0.382 |
| 6 | 3.68 | 2 | 0.159 |  | 2.43 | | 2 | | 0.296 |  | 0.04 | 2 | 0.978 |  | 4.97 | 2 | 0.083 |
| 7 | 2.47 | 2 | 0.291 |  | 10.03 | | 2 | | **0.007** |  | 5.28 | 2 | 0.071 |  | 0.19 | 2 | 0.909 |
| 37 | 6.89 | 2 | 0.032 |  | 5.80 | | 2 | | 0.055 |  | 0.87 | 2 | 0.648 |  | 3.736 | 2 | 0.155 |
| 27 | 1.72 | 2 | 0.423 |  | n.a. | |  | |  |  | 6.54 | 2 | 0.038 |  | 2.99 | 2 | 0.224 |
| 8 | 7.57 | 2 | 0.023 |  | 0.18 | | 2 | | 0.912 |  | 0.16 | 2 | 0.922 |  | 0.50 | 2 | 0.777 |
| 28 | 2.86 | 2 | 0.240 |  | n.a. | |  | |  |  | 1.73 | 2 | 0.420 |  | n.a. |  |  |
| 39 | 2.36 | 2 | 0.307 |  | n.a. | |  | |  |  | 0.62 | 2 | 0.732 |  | n.a. |  |  |
| 9 | 13.19 | 2 | **0.001** |  | 5.57 | | 2 | | 0.062 |  | 0.03 | 2 | 0.982 |  | 4.249 | 2 | 0.120 |
| 10 | 0.85 | 2 | 0.652 |  | n.a. | |  | |  |  | 3.59 | 2 | 0.166 |  | 5.07 | 2 | 0.079 |
| 11 | 8.98 | 2 | 0.011 |  | 1.06 | | 2 | | 0.587 |  | 2.03 | 2 | 0.363 |  | 0.59 | 1 | 0.441 |
| 29 | 10.46 | 2 | **0.005** |  | n.a. | |  | |  |  | 9.69 | 2 | **0.008** |  | n.a. |  |  |
| 12 | 0.92 | 2 | 0.63 |  | n.a. | |  | |  |  | 0.84 | 2 | 0.657 |  | 2.84 | 2 | 0.241 |
| 13 | 4.85 | 2 | 0.088 |  | 3.03 | | 2 | | 0.220 |  | 5.56 | 2 | 0.062 |  | 3.40 | 2 | 0.182 |
| 30 | 2.84 | 2 | 0.241 |  | 3.24 | | 2 | | 0.198 |  | 1.47 | 2 | 0.479 |  | n.a. |  |  |
| 14 | 1.59 | 2 | 0.453 |  | 9.99 | | 2 | | **0.007** |  | 0.21 | 2 | 0.901 |  | 2.68 | 2 | 0.261 |
| 38 | 6.43 | 2 | 0.040 |  | n.a. | |  | |  |  | 7.10 | 2 | 0.029 |  | 2.26 | 2 | 0.322 |
| 15 | 12.21 | 2 | **0.002** |  | 9.50 | | 2 | | **0.009** |  | 0.34 | 2 | 0.842 |  | 5.49 | 2 | 0.067 |
| 16 | 1.56 | 2 | 0.459 |  | n.a. | |  | |  |  | 0.87 | 2 | 0.646 |  | 4.62 | 2 | 0.099 |
| 17 | 7.57 | 2 | 0.023 |  | 11.12 | | 2 | | **0.004** |  | 12.58 | 2 | **0.002** |  | 3.68 | 2 | 0.158 |
| 18 | 6.75 | 2 | 0.034 |  | 9.46 | | 2 | | **0.009** |  | 4.08 | 2 | 0.130 |  | 5.06 | 2 | 0.080 |
| 19 | 0.15 | 2 | 0.927 |  | 5.43 | | 2 | | 0.066 |  | 2.73 | 2 | 0.256 |  | 9.11 | 2 | **0.010** |
| 31 | 2.68 | 2 | 0.262 |  | n.a. | |  | |  |  | 9.13 | 2 | 0.010 |  | 2.69 | 2 | 0.261 |
| 40 | 3.49 | 2 | 0.175 |  | n.a. | |  | |  |  | 5.78 | 2 | 0.056 |  | n.a. |  |  |
| 32 | 4.35 | 2 | 0.114 |  | 5.10 | | 2 | | 0.078 |  | 0.50 | 2 | 0.778 |  | 3.42 | 2 | 0.181 |
| 33 | 3.46 | 2 | 0.177 |  | 8.68 | | 2 | | 0.013 |  | 2.60 | 2 | 0.056 |  | 2.16 | 2 | 0.340 |
| 20 | 1.26 | 2 | 0.532 |  | n.a. | |  | |  |  | 1.59 | 2 | 0.452 |  | 0.106 | 2 | 0.948 |
| 21 | 1.28 | 2 | 0.527 |  | 4.06 | 2 | | 0.131 | |  | 0.28 | 2 | 0.869 |  | 4.71 | 2 | 0.095 |
| 22 | n.a. |  |  |  | n.a. |  | |  | |  | 0 | 1 | 1 |  | n.a. |  |  |
| n.a. = not accessed | | | | | | | | | | | | | | | | | |
